# Supplementary material for: Folate Deficiency Enhanced Inflammation and Exacerbated Renal Fibrosis in High-Fat High-Fructose Diet-Fed Mice
Source: Nutrients. 2023 Aug 17;15(16):3616. doi: 10.3390/nu15163616 (PMC10458828; doi:10.3390/nu15163616)
Supplement: Supplementary file 1 [file nutrients-15-03616-s001.zip › nutrients-2549595-supplementary.pdf]

nutrients

MDPI

Supplementary Materials

Folate Deficiency Enhanced Inflammation and Exacerbated Renal Fibrosis in High-Fat High-Fructose Diet-Fed Mice

Table S1. Characteristics of eight-week-old C57BL/6 mice prior to the beginning of the experiment.

	NF+f (n = 11)	NF-f (n = 11)	HFF+f (n = 16)	HFF-f (n = 16)
**Body weight (g)**	23.3 ± 0.3	23.3 ± 0.3	23.3 ± 0.2	23.3 ± 0.2
**Fasting serum TC (mg/dL)**	84.2 ± 3.8	92.8 ± 2.7	86.2 ± 3.9	88.9 ± 2.5
**Fasting Serum LDL-C (mg/dL)**	22.0 ± 2.1	26.4 ± 1.9	21.5 ± 2.7	22.6 ± 1.4
**Fasting blood glucose (mg/dL)**	99.0 ± 3.4	105.0 ± 5.9	92.0 ± 3.8	100.0 ± 4.1
**Serum folate (ng/mL)**	40.7 ± 3.5	39.3 ± 2.0	42.6 ± 3.5	43.0 ± 2.2
**Serum homocysteine (μmol/L)**	1.11 ± 0.04	1.13 ± 0.05	1.11 ± 0.05	1.25 ± 0.07

Data are mean ± SEM (*n* = 11 or 16 per group). TC: total cholesterol; LDL-C: low-density lipoprotein cholesterol.

Table S2. Pearson correlation between serum cholesterol and renal functional or fibrotic indicators in mice fed NF or HFF diet for 12 months.

NF, HFF Diet		Serum TC	Serum LDL-C
**Renal type IV collagen**	r value	0.718	0.632
	p value	<0.0001	<0.0001
**Renal type I collagen**	r value	0.634	0.566
	p value	<0.0001	<0.0001
**Glomerulus size**	r value	0.688	0.599
	p value	<0.0001	<0.0001
**Renal fibrosis area**	r value	0.644	0.538
	p value	<0.0001	<0.0001
**Serum creatinine**	r value	0.588	0.477
	p value	<0.0001	<0.0001
**Urinary protein-to-creatinine ratio**	r value	0.466	0.353
	p value	<0.0001	0.010
**Urinary NGAL**	r value	0.320	0.356
	p value	0.018	0.010

NGAL: neutrophil gelatinase-associated lipocalin.

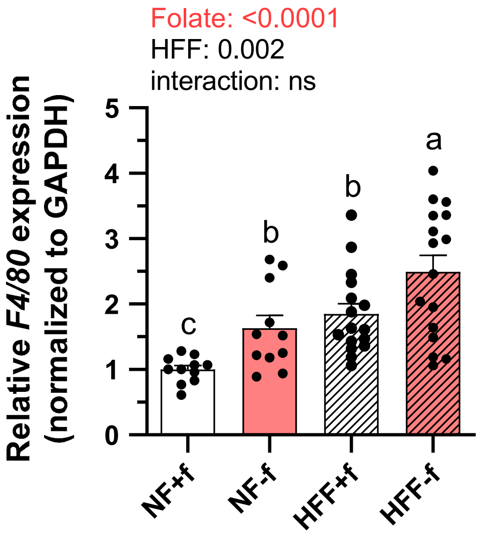

Figure S1. Both HFF diet and folate deficiency increased *F4/80* gene expression in the kidney. Renal *F4/80* gene levels were determined by qRT-PCR method. Data are mean ± SEM (n = 11 or 16 per group). Values were analyzed with two-way ANOVA with folate and HFF as independent factors, followed by Duncan's post hoc test. $p < 0.05$ or different letters was considered statistically significant. ns: not significant.
